# Pain Perception and Acceptance of Illness in Patients Undergoing Phacoemulsification Cataract Surgery under Drip Anesthesia

**DOI:** 10.3390/jcm8101575

**Published:** 2019-10-01

**Authors:** Krystyna Kowalczuk, Mateusz Cybulski, Łukasz Cybulski, Elżbieta Krajewska-Kułak

**Affiliations:** 1 Department of Integrated Medical Care, Faculty of Health Sciences, Medical University of Białystok, M. Skłodowskiej-Curie 7A str., 15-096 Białystok, Poland; 2 National Security student, Faculty of Social Sciences, University of Warmia and Mazury in Olsztyn, Żołnierska 14 str., 10-561 Olsztyn, Poland

**Keywords:** pain, pain perception, acceptance of illness, cataract, phacoemulsification

## Abstract

For many years, cataracts have been the main cause of vision loss and vision impairments in the world (43% and 33%, respectively). Currently, the most common surgical method for treating cataracts is phacoemulsification. The aim of this study was to assess the pain perception and acceptance of illness connected with awaiting phacoemulsification cataract surgery under intravenous drip anesthesia, as well as to determine the effect of selected sociodemographic factors on the above. Methods: The study was conducted in a group of patients of the Department of Ophthalmology, University Clinical Hospital in Białystok, Poland suffering from cataracts, who underwent phacoemulsification surgery under intravenous drip anesthesia. The study group consisted of 151 people. The study used an original short questionnaire and three standardized psychometric scales: The acceptance of illness scale (AIS), the Beliefs about Pain Control Questionnaire (BPCQ), and the Coping Strategies Questionnaire (CSQ). Results: The median overall AIS point value was 24 points, which is considered an average score in terms of disease acceptance. Respondents assessed the influence of individual factors on the level of perceived pain and the impact of individual strategies for coping with pain similarly. The level of perceived pain decreased with the patient’s age. People with a higher education level experienced a greater level of pain; however, this relationship was not statistically significant. The place of residence did not affect the level of pain experienced during the procedure. Women had a greater level of acceptance of illness. The respondents’ education level negligibly differentiated the approach to the disease. The place of residence also did not affect the assessment of illness acceptance as measured by the AIS. Whether the surgery pertained to the first or second eye did not significantly affect the approach to the disease. Conclusions: The level of acceptance of illness and pain perception were at a moderate level among the patients. The acceptance of illness was significantly influenced by the age of patients and the waiting period for phacoemulsification, and this level of acceptance decreased significantly with the increasing age of patients. The longer the waiting period for surgery, the lower the acceptance of illness. Sex significantly differentiated the level of pain experienced during the procedure. Education and place of residence did not significantly affect the acceptance of illness and the feeling of pain.

## 1. Introduction

For many years, cataracts have been the main cause of vision loss and vision impairments in the world (43% and 33%, respectively) [1]. The incidence of the disease increases with age, from less than 5% in people under 65 to around 50% in people aged 75 years and older, which is why it is referred to as an aging-related problem [2]. Epidemiological studies on cataract risk conducted over the last few decades have shown that the development of this disease depends on many factors in the elderly [3].

Currently, the most common surgical method used to treat cataracts is phacoemulsification. This consists of breaking down the cloudy lenses with the help of ultrasound, and removal of the resulting broken fragments. Phacoemulsification can be used at any stage of cataract development. In place of the removed lenses, a lens is implanted with appropriately selected optical parameters. The advantages of this method include being able to perform the procedure under intravenous drip anesthesia, short duration, and lack of need for hospitalization [4].

Vision disorders are associated with limitations of mobility, everyday activities, and physical acuity [5]. Loss of vision and vision impairment are not only health problems, sensu stricto, but also important determinants of all aspects of life of this social group, particularly their quality of life [6,7].

In recent years, there has been rapid development in the research on mutual interactions between pain as a physiological process and its perception by the individual [8,9,10]. Pain is characterized by two aspects. The first is the sensory function, which is directly responsible for the sensation of pain. Therefore, it is possible to locate the site of pain. The second is the emotional aspect, which is characterized by a psychological response to a painful stimulus. The emotional component of pain is usually a subjective sensation, so pain varies between individuals, especially in older people [11,12,13]. Significant determinants of feeling pain, in psychological terms, include a sense of control over the pain and self-efficacy [14].

Adaptation to and acceptance of illness play an important role in both the control and self-control of diseases. Acceptance of a given disease greatly affects a person’s self-esteem and ability to adapt to existing limitations which, in turn, determine the subjective quality of life [15]. The literature on the subject [16,17] has demonstrated that a higher acceptance of illness is related to weaker negative reactions and emotions associated with the disease and treatment.

The aim of the conducted study was to assess pain perception and acceptance of illness after phacoemulsification cataract surgery under intravenous drip anesthesia, as well as to determine the effect of selected sociodemographic factors (sex, age, level of education, place of residence, waiting time for surgery, and eye operation) on the above. An analysis of the literature did not uncover any publications on similar subjects in ophthalmology. It was assumed that the perception of pain after phacoemulsification was at the level of three to five points on the numerical rating scale (NRS). In addition, we surmised that the degree of acceptance of the illness in the group of patients with cataracts would be moderate and comparable to the degree of acceptance of other somatic diseases, such as diabetes, respiratory diseases, and cardiovascular diseases.

## 2. Materials and Methods

### 2.1. Participants

The study was conducted in a group of patients of the Department of Ophthalmology, University Clinical Hospital in Bialystok, suffering from cataract, who underwent phacoemulsification surgery under intravenous drip anesthesia. The study group consisted of 151 people. Men accounted for almost half of the analyzed population. The largest group of respondents (every third studied person) were patients aged 71–80. Every fifth patient was over 81 years old. Among the respondents, the majority had a vocational education (almost 40%), every sixth respondent completed high school, and 23% had a higher education. In the studied group, the vast majority of people were professionally inactive, 66% were retired, and every fifth person received a pension. More than half of the studied patients were married, and almost every third respondent had lost his/her spouse. The percentage distribution of inhabitants of villages and cities was almost even. The vast majority of the respondents lived with their family (spouse or children). Only 14% of the respondents lived alone, and less than every tenth stayed with a caretaker. The respondents’ detailed sociodemographic data characteristics are shown in Table 1. Due to the fact that a significant percentage of the patients did not provide all the answers to the analyzed questionnaire, the table below also includes the percentage of responses without missing data (these percentages are given in brackets).

Criteria for inclusion in the study included written consent to participate in the study, in addition to having phacoemulsification cataract surgery under drip anesthesia in the course of one-day hospitalization. Each participant could withdraw from the study at any time.

Participant selection was intentional. For the study to be representative, the authors aimed to collect at least 150 fully completed questionnaires. More copies of the research tool were distributed (283); however, not all of the questionnaires were returned. A significant percentage of the people included in the study resigned from participating while completing the questionnaire. The response rate was 53.36%, and the attrition rate was 46.64%.

The study was conducted from February to June 2019. The respondents received paper questionnaires, which they filled in with the help of an interviewer (a member of the research team) in the hospital up to two hours after the phacoemulsification procedure was completed, after providing detailed instructions for completing the questionnaires. The research conforms with the Good Clinical Practice guidelines, and the procedures followed were in accordance with the Helsinki Declaration of 1975, as revised in 2000 (concerning the ethical principles for the medical community and forbidding release of the patient’s name and initials, or the hospital evidence number). The study was reviewed and approved by the Bioethics Committee of the Medical University in Białystok (statute no. R-I-002/232/2016).

### 2.2. Measures

The study used an original short questionnaire and three standardized psychometric scales: The acceptance of illness scale (AIS) by B. J. Felton, T. A. Revenson, and G. A. Hinrichsen, translated into Polish by Z. Juczyński; the Beliefs about Pain Control Questionnaire (BPCQ) by S. Skevington, translated into Polish by Z. Juczyński; and the Coping Strategies Questionnaire (CSQ) by A. K. Rosenstiel and F.J. Keefe, translated into Polish by Z. Juczyński.

#### 2.2.1. Original Questionnaire

The original questionnaire consisted of eight closed-ended single-answer and multiple-choice questions. The questions concerned coexisting eye diseases; coexisting systemic diseases; the time of the decision to undergo cataract surgery from the moment of diagnosis, expressed in years; the period of waiting for surgery after enrolling in the queue, expressed in months; information about which eye was operated on and the place where the surgery was performed; and sources of information about the anesthesia used during the cataract surgery.

The questionnaire also asked about the severity of pain during the use of drip anesthesia. The numerical rating scale was used for this purpose. The NRS is easy to use and has been shown to have a high sensitivity and reliability compared with other scales employed for pain measurement. It contains 11 degrees of pain intensity, from 0 to 10, where 0 means a complete absence of pain and 10 indicates the worst imaginable pain. This scale is characterized by a significant reproducibility of results and is useful in scientific applications. Due to the fact that it is easy to understand for patients and easy to use, it is currently recommended in clinical practice for both acute and chronic pain [18].

#### 2.2.2. Acceptance of Illness Scale

AIS statements express certain difficulties and limitations resulting from health status. The level of acceptance of an illness is shown through a lack of negative reactions and emotions associated with the disease. The AIS can be used to measure the acceptance of any disease. The scale has eight questions describing the negative consequences of poor health pertaining to the limitations imposed by the disease, lack of self-sufficiency, feeling of dependence on others, and reduced self-esteem. In each statement, the respondent determines his/her current state using the five-point Likert scale (from 1 “I definitely agree” to 5 “I strongly disagree”). Definite agreement (one point) expresses bad adaptation to the disease and strong psychological discomfort, while disagreement (five points) indicates acceptance of the disease. The overall score ranges from eight to 40 points. The higher the score, the greater the acceptance of the condition and the less negative emotions associated with the disease. A low score is considered to be less than 20 points, and values above 30 points signify a high level of acceptance of one’s disease, while an average score ranges from 20 to 30 points. Interpretation of the obtained results also depends on the average acceptance rates in different groups of patients. The Cronbach’s alpha for the method is 0.82 [19,20].

#### 2.2.3. The Beliefs about Pain Control Questionnaire

The BPCQ refers to scales measuring the localization of control, including health control. It is used to assess the strength of individual beliefs regarding pain control by means of the following:

1. Internal factors;
2. The influence of doctors (the power of others);
3. Random events [19,21].

The Cronbach’s alpha for the whole scale is 0.75, and for subscales, the influence of doctors is 0.86, the internal localization of pain control is 0.82, and the influence of random events is 0.58. The BPCQ is designed to examine adult patients complaining of pain. It can also be used to measure beliefs about pain control in people who currently do not complain of pain. The questionnaire can be used in the diagnosis and treatment of patients with pain, whether treated in the hospital or as outpatients. The questionnaire consists of assessing the content of the given statements on a six-point Likert-type scale:

1. No, I completely disagree;
2. I disagree;
3. I rather disagree;
4. I rather agree;
5. I agree;
6. Yes, I completely agree [19,21].

The results cannot be presented as a single indicator. The sum is calculated separately for each dimension of the location of pain control. The range of possible points is from five to 30 for the internal control and from four to 24 for the other two dimensions. A higher score reflects stronger beliefs that the pain can be controlled by the influence of one factor [19,21].

#### 2.2.4. The Coping Strategies Questionnaire

The CSQ contains 42 statements describing different ways of coping with pain, as well as two questions about the assessment of one’s own coping skills and pain reduction. The methods of coping with pain reflect six cognitive strategies and one behavioral strategy which, in turn, are part of the three following factors:

- Active coping (re-evaluation of pain sensations, ignoring sensations, declaring coping);
- Diverting attention and taking substitute actions (diverting attention and increased behavioral activity);
- Catastrophizing and hoping (catastrophizing and praying/hoping) [19,22].

The evaluation uses a seven-point Likert-type scale. A result ranging from 0 to 36 points is calculated for each strategy. The higher the score, the more important the method for coping with pain. In the case of questions about the degree of mastery and the possibility of reducing pain, the results range from zero to six. The higher the score, the more important are one’s own coping skills and the ability to reduce pain [19,22].

The questionnaire is used to assess the strategies for coping with experienced pain and their effectiveness in controlling and reducing pain, and is intended for examining adult patients complaining of pain in an outpatient or hospital setting. Previous studies have confirmed that the CSQ is a reliable and accurate measurement tool, and the psychometric characteristics of the Polish version of the questionnaire are satisfactory. The reliability was measured by Cronbach’s alpha as 0.80. A comparison of CSQ results with those of BPCQ has been shown to confirm the theoretical validity [19,22].

### 2.3. Statistical Analysis

Data were prepared using Microsoft Excel 2013 and statistical analysis was conducted using STATISTICA 13.3 software. The results are presented in the form of a summary of descriptive statistics in the compared groups (arithmetic mean, standard deviation, median, lower and upper quartile, and minimum and maximum values). Two groups were compared using Mann-Whitney’s test, and in case of three groups, the Kruskal-Wallis test was used. In addition, the chi-square test of independence was also used. The selection of a nonparametric test resulted from a fairly significant asymmetry in the distribution of pain intensity, as described in detail in the Results section. The results were considered statistically significant at *p* < 0.05.

## 3. Results

### 3.1. Characteristics of the Treatment

In the discussed group, in addition to the treated cataract, every third person suffered from glaucoma, and a slightly lower percentage (28%) from inflammatory diseases. Additional diseases coexisting with eye diseases most often included diabetes, hypertension, and atherosclerosis. Detailed data are presented in Table 2.

Half of the respondents decided to undergo cataract treatment within one year of diagnosis. The waiting period for surgery was up to half a year for every second patient, up to one year for every fifth person, and over a year also for around every fifth person (Table 3).

Almost two-thirds of the respondents (62.3%) had undergone surgery of the first eye, and the remaining patients (35.7%) had undergone surgery of the second eye. Three people (2.0%) did not answer the question.

The vast majority of phacoemulsification procedures (92.3%, n = 132) had been performed at the Department of Ophthalmology of the University Clinical Hospital in Białystok. The remaining 7.7% of respondents (n = 19) had undergone treatment at other medical facilities.

For the majority of the studied patients, medical personnel in the form of doctors and nurses were the main sources of information about anesthesia (Table 4).

Table 5 presents the characteristics of the pain experienced by patients under intravenous drip anesthesia. The results were scaled in such a way that the higher values correspond to a greater intensity of pain. It is worth noting that every third person reported the highest possible pain intensity value. Answers were obtained for 149 out of 151 respondents.

### 3.2. Acceptance of illness scale

**Figure 1:**
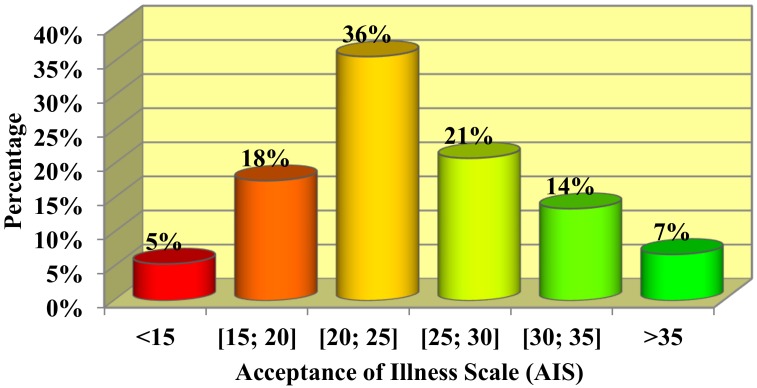
Percentage of patients with five-point ranges of values on the acceptance of illness scale (AIS).

The median overall AIS point value was 24 points, which is considered an average score in terms of disease acceptance (Table 6). Almost one-quarter of the respondents (22.3%, n = 33) did not accept their illness, 57.4% (n = 85) accepted it to a medium degree, and 20.3% (n = 30) showed a high level of acceptance. Figure 1 presents the percentage of people with five-point ranges of values on the AIS. The highest percentage of people (36.0%) were in the range of 20–25 points.

### 3.3. Possibilities of Pain Control–BPCQ

Respondents assessed the influence of individual factors on the level of perceived pain similarly, and the median value of the impact of all three measures on pain control on the BPCQ scale was 40 points (Table 6). The analysis below presents normalized values (scaled to a range of 0–100 points) because they are easier to interpret and allow a comparison of the importance of internal pain control with the other two factors. The average and other descriptive statistics were almost identical for individual BPCQ components.

### 3.4. Methods of Pain Control–CSQ

As with the BPCQ, respondents assessed the impact of individual strategies for coping with pain similarly. The highest median value (17 points) was obtained in the case of praying/hoping, and the lowest was obtained in the case of increased behavioral activity. Table 6 presents the values of selected descriptive statistics.

### 3.5. The Effect of Selected Sociodemographic Characteristics on the Level of Perceived Pain

Sex quite clearly differentiated the level of perceived pain during surgery (*p* = 0.0069). It was significantly higher in the group of women by 1.5 points, on average. The maximum pain level was felt by about every fourth man and almost every other woman.

The level of perceived pain decreased with the patient’s age, which resulted from the analysis of descriptive statistics, mainly the average and median. However, the difference between the groups was not so large that these conclusions could be considered fully reliable (the test probability value of *p* obtained using the Kruskal–Wallis test was more than 0.05).

People with a higher education level experienced a greater level of pain; however, this relationship was not statistically significant. The place of residence did not affect the level of pain experienced during the procedure.

The effect of waiting time for treatment on the subjective feeling of pain was close to the level of statistical significance, but the nature of this effect was complex because a higher level of pain was experienced by those waiting for the procedure for both relatively short and relatively long periods.

People who were operated on for the first time experienced significantly higher pain than patients who had undergone surgery of the second eye (on average, about 7.6 points versus 6.2 points, respectively).

A detailed summary of the effect of selected sociodemographic characteristics on the occurrence of pain with the use of drip anesthesia, taking into account selected descriptive statistics and *p* values, is presented in Table 7.

### 3.6. The Effect of Selected Sociodemographic Characteristics on the Acceptance of Illness Level

Women had a greater level of acceptance of illness. The difference between sex, both in relation to the numerical values (test probability value of *p* obtained with the Mann–Whitney test was 0.0626) and the adjective classification (probability value of *p* obtained with the chi-square test was 0.0750), was close to statistical significance.

The level of acceptance of illness dropped significantly with the patients’ age. This was particularly visible on the more accurate, numerical AIS. The percentage distribution of illness acceptance level was not as clearly differentiated as the original numerical values. 

The respondents’ education level almost completely did not differentiate the approach to the disease. The place of residence also did not affect the assessment of illness acceptance as measured by the AIS.

The longer the waiting time for the procedure, the lower the acceptance of illness, and this correlation was statistically significant. However, we did not observe this after categorizing the AIS values; the transition to a scale ranking the acceptance of illness resulted in the loss of information and also the clear, statistically significant relationship. 

Whether the surgery pertained to the first or second eye did not significantly affect the approach to the disease (*p* = 0.1680).

A detailed summary of the impact of selected sociodemographic characteristics on the level of acceptance of illness in relation to numerical values, including selected descriptive statistics and *p* values, is presented in Table 8. Table 9 presents a summary of selected sociodemographic characteristics on the level of acceptance of illness in relation to the degree of acceptance taking into account selected descriptive statistics and *p* values.

## 4. Discussion

In reviewing scientific databases of the available literature on pain perception and acceptance of illness by patients with cataracts, we did not find papers on this topic. Therefore, the results of our research were compared with results from the studies of other diseases. We believe that the results of this study will be significant for improving the quality of patient care and will be an important element in assessing the treatment process of patients with cataracts after surgery. The presented article may constitute the basis for planning activities aimed at sensitizing healthcare professionals in the discussed area and increasing the professional attitude of these employees towards patients with cataracts. Therefore, it seems advisable to conduct research that takes into account the assessment of pain perception and degree of acceptance of illness by patients with cataracts.

### 4.1. Acceptance of Illness Scale (AIS)

Our study showed that the respondents’ acceptance of the disease was average. The majority of respondents accepted their illness to a medium degree, which was reflected in the average level of the AIS results (the highest percentage of respondents assessed the acceptance of illness in the range of 20–25 points). Van Damme-Ostapowicz et al. [23] presented quite different results in their study, which included 140 malaria patients treated at the Madonna University Teaching Hospital in Elele (Nigeria). The median value of the AIS in that study was only 10.5 points, indicating that malaria was not accepted. Such large differences could be caused by the severe course of the disease in comparison with cataracts, the high risk of serious malaria complications, as well as sociodemographic factors such as the patients’ financial situation, in addition to the quality of the provided healthcare services.

Interestingly, the mean point value on the AIS among breast cancer patients in studies conducted by Polish authors was significantly higher than in our study [24,25]. A higher mean point value was also recorded among 117 patients on dialysis, with an average age of 29.5 years (25.32 points) in a study by Adamczuk et al. [26]. In this case, the higher level of illness acceptance—in comparison to the group included in our study—could be due to the younger age of the patients as well as the large percentage of patients that had prior kidney transplantation, which significantly reduces the burden associated with the disease. 

Patients with ulcerative colitis had an even higher mean value of illness acceptance using the AIS [27]. The was an average level of illness acceptance, and the mean point value on the AIS was 29.65 points. Similarly, a higher mean point value on the AIS was demonstrated by patients with lung diseases, such as asthma, chronic obstructive pulmonary disease, and obstructive sleep apnea (26.1 points) [28], than the cataract patients in our study.

An assessment of illness acceptance using the AIS, conducted among patients with chronic obstructive pulmonary disease by Uchmanowicz et al. [29], showed that the respondents were mainly characterized by a moderate level of acceptance, with a tendency to not accept their disease (20.6 points). In this case, the average value obtained on the AIS was lower than in our study. Cybulski et al., who studied people over 60 years of age, showed that the average level of acceptance of illness was 26.21 points. Rosińczuk et al. obtained similar point values for the acceptance of Parkinson’s disease in a group of 50 patients (25.28 points) [30]. Sierakowska et al. obtained almost identical results in their study [31]. The average level of illness acceptance in a group of patients with osteoarthritis was 25.75 points, which indicates an average level. Nevertheless, it should be emphasized that the compared groups differed in size, as well as in the diseases they had. 

The statistical analysis conducted in our study did not show a correlation between sex and acceptance of illness. The study by Chrobak-Bień et al. also reached the same conclusion [27]. These results are also in line with other results that did not confirm a higher level of acceptance of illness by men or women [32]. The analysis carried out by Staniszewska et al. [33] also did not show a statistically significant correlation between the patients’ sex and the level of acceptance of illness. In studies performed on surgery ward patients, an inverse correlation was found. According to these studies, men felt less psychological discomfort due to illness [34].

The statistical analysis also included age. Our study indicated a statistically significant negative correlation between age and the level of average values for the AIS. The older the patients, the lower the average level of illness acceptance. Cipora et al. [25] and Sierakowska et al. [31] demonstrated an identical correlation in their studies. Chrobak-Bień et al. [27] and Staniszewska et al. [33], however, did not find an effect of age on the acceptance of illness. Similarly, no such correlation was observed by Polish researchers from Toruń who conducted an analysis of women treated for pathological cervical lesions [35], nor by a research team from Rzeszów studying women after mastectomy [36]. Glińska et al. [32] determined that older patients adapted better to living with inflammatory bowel disease. The authors attributed this phenomenon to the more financially stable economic situation and professional status of older people. This observation was not confirmed by our research.

When studying the effect of education level on the acceptance of illness, no statistically significant correlation was found. Chrobak-Bień et al. [27] showed that patients with a higher education showed a higher level of acceptance of illness than people with a secondary or vocational education. Andrzejewska et al. [37] also showed higher point values of illness acceptance among people with a higher education. Glińska et al. [32] described the strong influence of higher education on the acceptance of illness. We noted a completely different correlation in our study. Data from the study of Cipora et al. [25] showed that women with a higher education had a higher level of illness acceptance compared with respondents with a lower level of education. The average AIS values did not differ significantly (*p* = 0.12). An analysis of the results of the study by Staniszewska et al. [33] did not show a significant correlation between illness acceptance and the respondents’ education. Similarly, Kaźmierczak et al. [35], who studied disease acceptance in women treated for pathological cervical lesions, and Pawlik et al. [36], who studied cancer acceptance in women after mastectomy, did not observe any effect of education level on illness acceptance. However, Niedzielski et al. [17] and Szafraniec et al. [38], who examined illness acceptance in patients with rheumatoid arthritis, obtained a negative level of acceptance of illness in people with a lower level of education. People with a higher education are characterized by a higher awareness of the risks associated with noncompliance with medical recommendations, and are, thus, more likely to learn to cope with the disease, which results in a greater acceptance.

In our study, we did not find any statistically significant differences in the level of illness acceptance depending on the place of residence. Cipora et al. noted a similar relationship [25]. However, it should be noted that urban residents had a slightly higher level of illness acceptance than patients from rural areas.

### 4.2. Perception of Pain

Patients have different strategies for reducing pain. Our study showed that the respondents’ perception of pain was average. Most of the respondents assessed the effect of all three measures on pain control included in the BPCQ similarly. The BPCQ results in the study by Czerw et al. [24] showed that patients with breast cancer mainly believed that doctors had the greatest influence on pain control and internal factors had the least influence. In addition, the same authors showed that in the case of pain management strategies, breast cancer patients most often preferred a strategy of coping with it, and least often, catastrophizing [24]. Rosenstiel and Keefe [22] and Juczyński [39] drew similar conclusions. We obtained completely different results in our study of cataract patients; the most frequently adopted strategy was praying/hoping, while the rarest was increased behavioral activity. Similar results in the case of praying/hoping, as one of the most common strategies for coping with pain, have been noted in many other publications studying cancer patients [40,41,42,43,44].

In the case of older people, Cybulski et al. [45] showed that the influence of internal factors and doctors on pain control in the BPCQ was higher than in our study among patients with cataracts, whereas in the case of random events, the same result was obtained. In a group of 100 patients with peripheral artery disease in the study by Kadłubowska et al. [46], the median of the influence of internal factors was 18 points, the influence of doctors was 19 points, and the effect of chance events was 16 points. The same study also included 100 patients with rheumatoid arthritis. In this group of patients, all three groups of factors affecting the perception of pain scored lower than those in patients with peripheral artery disease (internal factors 14 points, doctors 16 points, random events 15 points).

Our research showed that sex statistically significantly differentiated the level of pain experienced during the procedure. It was significantly higher in the group of women. However, there was no significant correlation between the level of pain and the patients’ age. Cybulski et al. [45] found no statistically significant differences between men and women, as well as between age groups in terms of the point values of individual scales and their subscales. In the study by Krajewski et al. [40], patients’ age also did not have a significant impact on the results of the CSQ, although there are studies in the literature in which age statistically significantly influenced the strategies for coping with pain [47].

Our study showed that people who were better educated perceived pain as more severe; however, there was no statistically significant correlation in this case. Krajewski et al. came to the same conclusions in their study [40], while Czerw et al. obtained completely different results [24,41]. The correlation we obtained may be illusory because women and people from younger age groups were better educated. To answer the question about the real impact of education, it would be necessary to perform multivariate analysis, e.g., regression analysis or analysis of variance, but the asymmetric distribution of the pain rating scale causes some difficulty, which makes it impossible to fully apply these analytical techniques.

When analyzing the place of residence, we found that patients in rural areas had significantly higher pain reduction abilities compared with patients living in urban areas. Krajewski et al. drew similar conclusions [40]. However, these results were in contrast to the results of other authors [24,43].

The effect of the waiting time for treatment on the subjective feeling of pain was close to the level of statistical significance, whereas the nature of this effect was complex. A higher level of pain was felt by people waiting for surgery for periods that were both relatively short (perhaps due to the course of the disease, they qualified for surgery faster, and this could have been related to the level of pain) and relatively long (perhaps the “fatigue” of waiting for the procedure and, thus, a negative evaluation of its course were at play here).

The conducted study showed that the respondents experienced more pain during the operation of the first eye than the second eye. Gayadine-Harricham et al. [48] reported similar results. Out of 69 patients enrolled in the study, only 13 people (19%) experienced more pain after the second-eye surgery. Shi et al. [49] obtained completely different results in their meta-analysis, which examined eight published studies. The authors showed that patients experienced a greater pain intensity during surgery of the second eye than during first-eye surgery. This fact may be associated with various types of anxieties after first-eye surgery, as well as previous experiences during the first operation. The differing results obtained in our study could also be due to the fact that the vast majority of respondents were people who had already undergone surgery of the first eye.

### 4.3. Impact of Conducted Research on the Quality of Patient Care and their Clinical Significance

The study showed that a longer waiting time for surgery enhances the perception of pain, and first-eye surgery is felt to be more painful than second-eye surgery. Therefore, in order to improve the quality of patient care, the waiting time for first-eye surgery should be reduced as much as possible, while the waiting period for second-eye surgery may be a little longer. 

Clinically, the study showed that the ideal solution would be a controlled administration of anesthetic drugs during phacoemulsification, depending on the patient’s pain. Currently, at the University Clinical Hospital in Białystok, anesthetics are administered only immediately before the procedure, which may affect the level of pain sensation after the surgery.

### 4.4. Limitations

This study had some limitations that need to be considered. They include the cross-sectional nature of the study and the use of only self-report questionnaires. Another limitation of our study was that the group of patients was too small. The sample size, which included data from a clinic located in one hospital, is too small to generalize conclusions that are representative of all patients with cataracts. However, this study could be a starting point for further research on the effect of sociodemographic variables on the acceptance of an illness and the pain perception in patients with cataracts after phacoemulsification. For this purpose, longitudinal studies are justified.

## 5. Conclusions

The studied group was characterized by an average level of acceptance of the disease and the perception of pain. Patient age and the waiting time for phacoemulsification cataract surgery significantly affected the acceptance of illness. The level of acceptance of illness dropped significantly with the patient’s age, and the longer the waiting period for the procedure, the lower the acceptance of illness. Respondents’ sex and whether the operation pertained to the first or the second eye had a statistically significant effect on the perception of pain. Sex quite clearly differentiated the level of pain experienced during the procedure, and the patients who were operated on for the first time had significantly more pain complaints than patients who had undergone a second surgery. No significant relationships were found between the education level and place of residence and the level of acceptance of illness and perception of pain.

## Figures and Tables

**Table 1:** Sociodemographic characteristics of the studied group of patients.

Sociodemographic Characteristic		Number	Percent
Sex	Female	54	35.8% (42.5%)
	Male	73	48.3% (57.5%)
	no response	24	15.9%
Age (years)	30–39	1	0.7%
	40–50	7	4.6%
	51–60	21	13.9%
	61–70	38	25.2%
	71–80	52	34.4%
	81–90	32	21.2%
Education	Primary	34	22.5% (22.8%)
	Vocational	57	37.7% (38.3%)
	Secondary	24	15.9% (16.1%)
	Higher education	34	22.5% (22.8%)
	No response	2	1.3%
Professional activity	Unemployed	1	0.7%
	Employed	18	11.9%
	Retired	101	66.9%
	Pensioner	31	20.5%
Marital status	Married	89	58.9%
	Single	12	7.9%
	Widowed	47	31.1%
	Divorced	3	2.0%
Place of residence	Village	71	47.0% (49.3%)
	City	73	48.3% (50.7%)
	No response	7	4.6%
People living together	Husband/Wife	83	55.0% (58.0%)
	Children	27	17.9% (18.9%)
	Caregivers	12	7.9% (8.4%)
	Alone	21	13.9% (14.7%)
	No response	8	5.3%

**Table 2:** Other eye diseases and systemic diseases in the studied group of patients.

**Eye diseases**	**Disease**	**Number**	**Percent ^1)^**
	Glaucoma	48	31.8%
	Inflammatory diseases	42	27.8%
	Retinal detachment	4	2.6%
	Macular degeneration	2	1.3%
	Other	3	2.0%
	No other diseases	56	37.1%
**Systemic disease**	Diabetes	92	60.9%
	Hypertension	81	53.6%
	Atherosclerosis	53	35.1%
	Depression	48	31.8%
	Heart failure	44	29.1%
	Other	4	2.6%
	No selection	5	3.3%

^1)^ Sums do not have to add up to 100%, as response options were multiple choice.

**Table 3:** Duration of time to make the decision to undergo cataract surgery and waiting for the procedure in the studied group of patients.

**Diagnosis of cataract and the decision to undergo surgery (years)**	**Time**	**Number**	**Percent**
	0–1	66	43.7% (50.4%)
	2–3	50	33.1% (38.2%)
	4–5	13	8.6% (9.9%)
	5–6	2	1.3% (1.5%)
	No response	20	13.2%
**Waiting time for surgery (months)**	1–6	79	52.3% (54.9%)
	7–12	30	19.9% (20.8%)
	13–18	30	19.9% (20.8%)
	19–25	5	3.3% (3.5%)
	No response	7	4.6%

**Table 4:** Source of information on surgical anesthesia.

Source of Information on the Topic of Anesthesia	Number	Percent
Doctor	48	31.8% (34.3%)
Nurse	39	25.8% (27.9%)
Family	21	13.9% (15.0%)
Friends	23	15.2% (16.4%)
No one	9	6.0% (6.4%)
No response	11	7.3%

**Table 5:** Intensity of pain experienced by patients under drip anesthesia.

Pain Experienced under Drip Anesthesia	Number	Percent
1	13	8.7%
2	2	1.3%
3	6	4.0%
4	11	7.4%
5	7	4.7%
6	12	8.1%
7	21	14.1%
8	16	10.7%
9	14	9.4%
10	47	31.5%

**Table 6:** Descriptive statistics for AIS, Beliefs about Pain Control Questionnaire (BPCQ), and Coping Strategies Questionnaire (CSQ).

Scale	Feature	*N*	*x*	Me	*SD*	*Q_1_*	*Q_3_*	Min.	Max.
AIS (pts)	Acceptance of disease	148	23.9	24	6.1	20	28	12	36
BPCQ (0–100 pts)	Internal	150	43.8	40	23.3	28	60	0	100
	Influence of doctors	150	44.4	40	23.2	25	60	0	100
	Random events	150	44.2	40	23.2	30	60	0	100
CSQ (pts)	Diverting attention	149	14.5	15	8.5	7	21	0	33
	Reinterpretation of pain sensations	149	14.9	15	8.8	7	22	0	35
	Catastrophizing	149	14.8	14	8.8	7	21	0	34
	Ignoring activities	149	15.6	16	9.9	6	23	0	35
	Praying/Hoping	149	15.8	17	9.7	7	24	0	36
	Coping self-assessment	149	15.1	15	9.0	7	21	0	34
	Increased behavioral activity	149	13.4	11	9.3	6	20	0	35

AIS—Acceptance of Illness Scale; BPCQ—The Beliefs about Pain Control Questionnaire; CSQ—Coping Strategies Questionnaire; Max—maximum; Me—median; Min—minimum; SD—standard deviation; pts—points; Q_1_—first quartile; Q_3_—third quartile; *x*—mean.

**Table 7:** Effect of selected sociodemographic characteristics on the occurrence of pain with the use of intravenous drip anesthesia.

Sociodemographic Characteristic		Pain Experienced under Drip Anesthesia					
		*N*	*x*	Me	*SD*	*Q_1_*	*Q_3_*
Sex	Female	54	8.13	9	2.29	7	10
	Male	71	6.63	7	3.14	4	10
	*p*	0.0069 *					
Age (years)	Up to 60	28	8.07	8.5	1.94	7	10
	61–70	38	7.47	8	2.40	6	10
	71–80	52	6.96	8	3.12	5	10
	81–90	31	5.97	6	3.48	3	10
	*p*	0.1693					
Education	Primary	34	6.44	7	2.84	4	9
	Vocational	57	7.07	8	2.97	6	10
	Secondary	23	7.65	9	2.87	6	10
	Higher education	33	7.55	9	2.85	6	10
	*p*	0.2270					
Place of residence	Village	70	7.11	8	2.98	5	10
	City	72	6.88	7	2.87	5.5	9.5
	*p*	0.4639					
Waiting time for surgery (months)	1–6	79	7.25	8	2.93	6	10
	7–12	30	6.13	6.5	2.58	4	7
	>12	35	7.29	8	3.08	5	10
	*p*	0.0631					
Eye operation	First eye	94	7.59	8	2.54	6	10
	Second eye	54	6.19	6.5	3.29	4	10
	*p*	0.0152 *					

Me—median; *p*—*p*-value; SD—standard deviation; Q_1_—first quartile; Q_3_—third quartile; *x*—mean; *—statistically significant value.

**Table 8:** Effect of selected sociodemographic characteristics on the level of acceptance of illness with reference to numerical values.

Sociodemographic Characteristic		AIS (pts)					
		*N*	*x*	Me	*s*	*c*_25_	*c*_75_
Sex	Female	52	25.2	24.5	6.3	20.5	30
	Male	73	23.1	24	5.4	18	26
*p*		0.0626					
Age (years)	up to 60	29	25.4	26	5.7	23	30
	61–70	35	26.3	25	5.1	23	30
	71–80	52	21.8	21	6.1	18	24.5
	81–90	32	23.2	23	6.2	20	27.5
*p*		0.0007 *					
Education	Primary	34	24.6	24	5.9	18	29
	Vocational	54	23.4	24	5.2	20	27
	Secondary	24	24.2	24	7.0	19	28.5
	Higher education	34	23.3	23	6.8	20	28
*p*		0.8364					
Place of residence	Village	70	23.8	24	6.1	18	28
	City	71	24.1	24	6.2	21	27
*p*		0.8259					
Waiting time for surgery (months)	1–6	76	25.0	24.5	5.8	20.5	29.5
	7–12	30	23.3	22	6.1	18	28
	>12	35	22.0	22	6.7	18	25
*p*		0.0416 *					
Eye operation	First eye	91	24.4	24	5.9	20	29
	Second eye	54	23.0	22	6.5	18	27
*p*		0.1680					

AIS—Acceptance of Illness Scale; *p*—*p* value; pts—points; *—statistically significant value.

**Table 9:** Effect of selected sociodemographic characteristics on the level of acceptance of the disease with reference to the degree of acceptance.

Sociodemographic Characteristic		Degree of Acceptance of Disease			Total	*p*
		None	Average	High		
Sex	Female	8 (15.4%)	29 (55.8%)	15 (28.8%)	52	0.0750
	Male	19 (26.0%)	44 (60.3%)	10 (13.7%)	73	
Total		27	73	25	125	
Age (years)	Up to 60	4 (13.8%)	17 (58.6%)	8 (27.6%)	29	0.0572
	61–70	3 (8.6%)	23 (65.7%)	9 (25.7%)	35	
	71–80	19 (36.5%)	25 (48.1%)	8 (15.4%)	52	
	81–90	7 (21.9%)	20 (62.5%)	5 (15.6%)	32	
Total		33	85	30	148	
Education	Primary	9 (26.5%)	17 (50.0%)	8 (23.5%)	34	0.8339
	Vocational	11 (20.4%)	35 (64.8%)	8(14.8%)	54	
	Secondary	6 (25.0%)	12 (50.0%)	6 (25.0%)	24	
	Higher education	7 (20.6%)	20 (58.8%)	7 (20.6%)	34	
Total		33	84	29	146	
Place of residence	Village	19 (27.1%)	37 (52.9%)	14 (20.0%)	70	0.5765
	City	14 (19.7%)	42 (59.2%)	15 (21.1%)	71	
Total		33	79	29	141	
Waiting time for surgery (months)	1–6	13 (17.1%)	44 (57.9%)	19 (25.0%)	76	0.3567
	7–12	9 (30.0%)	15 (50.0%)	6 (20.0%)	30	
	>12	11 (31.4%)	19 (54.3%)	5 (14.3%)	35	
Total		33	78	30	141	
Eye operation	First eye	18 (19.8%)	51 (56.0%)	22 (24.2%)	91	0.3014
	Second eye	15 (27.8%)	31 (57.4%)	8 (14.8%)	54	
Total		33	82	30	145	
